# Attitudes towards sex workers: a nationwide cross-sectional survey among German healthcare providers

**DOI:** 10.3389/fpubh.2023.1228316

**Published:** 2023-09-06

**Authors:** Benedikt P. Langenbach, Andreas Thieme, Raquel van der Veen, Sabrina Reinehr, Nina R. Neuendorff

**Affiliations:** ^1^Department of Psychiatry and Psychotherapy, LVR University Hospital Essen, Faculty of Medicine, University of Duisburg-Essen, Essen, Germany; ^2^Center for Translational Neuro- and Behavioral Sciences (C-TNBS), University Hospital Essen, University of Duisburg-Essen, Essen, Germany; ^3^Department of Neurology, University Hospital Essen, University of Duisburg-Essen, Essen, Germany; ^4^Experimental Eye Research Institute, University Eye Hospital, Ruhr-University Bochum, Bochum, Germany; ^5^Department of Haematology and Stem Cell Transplantation, University Hospital Essen, Faculty of Medicine, University of Duisburg-Essen, Hufelandstr, Essen, Germany

**Keywords:** attitude, health care sector, health personnel, Germany, sex work, sex worker, social stigma

## Abstract

**Background:**

Worldwide, sex workers face stigmatization and discrimination, also within healthcare. Only few studies on healthcare providers’ attitudes towards care of sex workers have been performed. This study assessed attitudes and knowledge of healthcare providers in Germany towards sex workers and their specific health risks.

**Methods:**

German healthcare professionals and medical students were invited to participate in a nationwide cross-sectional study in 2022. The online survey used a German translation of the “Attitudes towards Prostitutes and Prostitution Scale” by Levin and Peled for assessment of attitudes towards sex work and workers, together with prevalence estimates of common mental and physical disorders.

**Results:**

A total of 469 questionnaires were included into analysis. Older participants tended to regard sex work as less of a choice (*p* < 0.004) and sex workers as more victimized (*p* < 0.001). The frequency of professional contact to sex workers neither affected the perception of sex workers’ status as victims vs. independent individuals, nor the perceived moral status. Moreover, healthcare professionals overestimated the prevalence of various disorders which was influenced by participants’ attitudes towards sex workers.

**Discussion:**

A comparison to a recent Allensbach survey demonstrated similar attitudes of healthcare providers and the general population towards sex workers. Our results suggest that German healthcare professionals are not free of prejudices against sex workers, as has been shown for other marginalized groups in society. Instead, they seem to be influenced by personal opinion rather than by objective facts which they should have acquired during their professional education. Future interventions (e.g., better training regarding marginal societal groups) are necessary to encounter these issues in order to improve healthcare for sex workers.

## 1. Introduction

A sex worker is defined as a person “who accepts money or goods for sexual services and who explicitly defines this as an income-generating activity, even if not considering sex work as his or her profession” (1). Attitudes towards sex work have always been controversial and are rather negative ranging from condemnation over tacit acceptance to compelling need. However, prejudice against sex workers predominates in most societies and often leads to discrimination and other human rights violations (2). It is likely that these circumstances also aggravate healthcare inequalities since utilization of healthcare services particularly depends on socio-economic aspects (3). Indeed, reduced utilization of healthcare has been shown for sex workers (4, 5). Given the existing prejudices in large parts of society, it is possible that these also are present in healthcare professionals. Negative experiences with healthcare providers, including anxiety associated with physical exams, discomfort with discussing sensitive topics or male doctors’ insensitivity to female sex workers’ need, are known barriers hindering healthcare utilization (6, 7). Consequently, negative attitudes towards sex workers and the anticipation of these negative attitudes by sex workers themselves may contribute to poor healthcare utilization and a reduced quality of medical care. However, research on healthcare professionals’ knowledge of and attitudes towards sex workers is sparse. Most evidence is based on surveys or interviews with sex workers themselves, rather than data acquired from healthcare providers (8). To the best of our knowledge, only two studies investigated the attitudes towards sex work in nursing and psychology students, respectively. It was shown that a feminist mindset corresponds to more negative attitudes towards sex work, that attitudes change during professional training, and that attitudes are related to willingness to care for sex workers (9, 10).

Generally, in psychology, an attitude refers to a set of emotions, beliefs, and behaviors towards a particular object, person, thing, or event (11, 12). It is suggested that there are distinct components that form attitudes. In 1998, Eagly and Chaiken described a tri-component ABC-model: **a**ffective component, **b**ehavioral, and **c**ognitive. In brief, the affective component refers to emotional reactions or feelings an individual has towards an object, person, issue, or situation, while the behavioral component refers to how the individual behaves, or acts based on their attitude. The cognitive component refers to the beliefs, thoughts, and attributes that an individual associates with an object, person, issue, or situation. Because attitudes can influence peoples’ behavior (13), it is important to be aware of healthcare professionals’ attitudes towards sex workers. If the prevalence of prejudices represents the population average, this may significantly discourage sex workers from seeking healthcare service. In addition to an exploratory description of healthcare professionals’ general views on sex workers, we built on existing knowledge in other areas of medical research to generate the following hypotheses: (I.) Similar to trends observed in the general population, we assumed that women would rate sex work rather negative compared to men, and (II.) that older participants would rate sex work as more negative than younger ones. Besides these demographic factors, it is known that contact with stereotyped populations generally reduces stereotypes (a process called the “contact hypothesis” in social psychology) (14). We therefore hypothesized that (III.) the frequency of professional contact with sex workers would influence the attitudes towards and the medical evaluation of sex workers.

Finally, some data suggest that healthcare professionals’ base their treatment decisions not only on their expertise, but also on their attitudes (15). Consequently, we expected a similar effect in our sample and expected that healthcare professionals’ estimates of sex workers’ health risks would be influenced by their attitudes. Specifically, we hypothesized that (IV.) participants who rate sex workers as victims expect that sex workers are at higher risks for various mental and somatic conditions.

In sum, our main aim was to provide a comprehensive view on healthcare providers’ knowledge of and attitudes against sex workers in Germany.

## 2. Methods

### 2.1. Study procedures

This nationwide, prospective, cross-sectional study collected data over a period of 90 days from August 8th to November 4th, 2022 with the aim of including as many eligible participants as possible. The survey was generated with SoSci Survey and provided on www.soscisurvey.de (16). Invitations to participate were disseminated through different social media platforms (Instagram and Facebook), via newsletters of universities and healthcare related societies (e.g., “Berufsverband Deutscher Internisten”/Association of German internists, “Deutsche Gesellschaft für Verhaltenstherapie”/German society for behavioral therapy), and personal contacts. Participation was restricted to German healthcare workers and medical students (for detailed information concerning the exact professions, see Table 1). We included all complete data sets of participants that stated to work in one of the professions of interest (healthcare workers with direct contact to patients). Incomplete surveys, and surveys of participants that did not have the required profession (e.g., lab personnel without contact to patients) were excluded from data analysis.

**Table 1:** Participants’ characteristics.

Participants’ characteristics	
Variable	Number (%)
Median Age ± SD (range), (*n* = 464)	34.2 ± 12.3 (18–85 years)
Age group (*n* = 464)	
<30 years	217 (43.1)
>30 years	182 (36.2)
>45 years	76 (15.1)
>60 years	28 (5.6)
Gender (*n* = 469)	
Female	334 (71.2)
Male	124 (26.4)
Indeterminate	11 (2.3)
Highest level of education (*n* = 469)	
No school-leaving certificate	1 (0.2)
Lowest school-leaving certificate “Hauptschule”	2 (0.4)
Intermediate school-leaving certificate “Realschule”	20 (4.3)
Advanced school-leaving certificate “Gymnasium”	189 (40.3)
Academic degree	169 (36.0)
PhD	88 (18.8)
Profession (*n* = 469)	
Physician	127 (27.1)
Nurse	72 (15.4)
Psychotherapist	66 (14.1)
Social worker	49 (10.4)
Medical student	154 (32.8)
Paramedic	1 (0.2)
Working place (*n* = 469)	
Hospital	300 (64.0)
Office	56 (11.9)
Counselling center	38 (8.1)
Others	76 (16.0)
Area of working place (*n* = 469)	
Village	7 (1.5)
Town (<30,000 residents)	19 (4.1)
Town (<100,000 residents)	47 (10.0)
City (<500,000 residents)	148 (31.6)
City (>500,000 residents)	248 (52.9)
How many sex workers do you care for per year? (*n* = 469)	
0	195 (41.6)
1–10	94 (20.0)
11–100	37 (7.9)
>100	19 (4.1)
Unknown	124 (26.4)

Participants participated voluntarily, did not report their name, and the information provided was not specific enough to identify individual participants. Still, the data were only accessible to the designated researchers, as required by the local ethics committee.

### 2.2. Composition of the survey

#### 2.2.1. Demographical data

Participants were asked to provide their age, gender, level of education, profession, working area (hospital, office, counselling center, others), and location of working place (city, town, or village). Furthermore, the frequency of professional contact with sex workers was recorded in five distinct categories (“I treated zero sex workers during the last year; one to ten sex workers; 11–100 sex workers; more than 100 sex workers; unknown”).

#### 2.2.2. Attitudes towards prostitutes and prostitution scale and general attitudes

To measure participants’ attitudes to both sex work and sex workers, we employed the “Attitudes towards Prostitutes and Prostitution Scale (APPS)” by Levin and Peled (17). It consists of 29 items (e.g., “prostitutes are victims of drug abuse,” “prostitution is trafficking of women,” “prostitution is a way to empower economically disadvantaged populations”). The questionnaire was translated from the original English version into German and then back translated by a professional translator and compared for consistency according to a standardized translation procedure for cross-cultural adaptation of instruments or scales for use in healthcare research (18). Instead of “prostitutes/prostitution” we used the term “sex worker/sex work” throughout the entire questionnaire (for the complete translation, see Supplement 1). The items of the APPS load onto four underlying factors: “prostitutes as normative vs. deviant” (in their personalities and behaviors); “prostitutes as choosing vs. victimized”; “prostitution as normativeness vs. deviance” (representing social normativeness vs. deviance); “prostitution as choice vs. victimization.”

To be able to compare the attitudes of our sample to that of the general population, we also added three items that closely resemble items from a representative study (19), see Supplement 2 for the complete items.

We also included questions about whether participants felt well-educated about sex work and sexual problems in their patients. Those data will be reported elsewhere.

#### 2.2.3. Estimation of disease prevalence among sex workers

To measure the estimates for prevalence of common mental and physical diseases in sex workers, participants were asked to estimate the point prevalence (0–100%) of anxiety, depression, post-traumatic stress disorder (PTSD), anal cancer, cervical cancer, and Human Immunodeficiency Virus (HIV) infection.

### 2.3. Ethical approval

The study conforms to the principles laid down in the Declaration of Helsinki. Ethical approval from the local ethics committee at the Medical Faculty of the University Duisburg-Essen, Germany, was obtained before any data was collected (ethics vote no.: 22-10678-BO). All participants gave their informed consent before participating in the survey.

### 2.4. Data analysis

Statistical analyses were computed using R (version 4.0.0) (20). We first evaluated the influence of age and gender on participants’ attitudes (measured with the APPS), using a (Welch) two-sample *t*-test for gender and the Pearson product–moment correlation for age (corresponding to hypotheses I and II). The number of participants who described their gender as “indeterminate” was small. Therefore, this group was excluded from analysis of gender impact. Age and gender were then included as covariates in the subsequent analyses.

To test for the influence of contact frequency on attitudes (hypothesis III), we ran four “analysis of variance” (ANOVA) tests following the general linear model with each of the four subscales of the APPS as dependent variable, frequency of professional contact with sex workers as predictor, and age and gender as covariates. In case of a significant result for frequency of professional contact (*p* < 0.05), post-hoc tests were calculated using Tukey’s honestly significant difference (HSD) test. Although ANOVAs are sufficiently robust against violations of normality in larger samples, we checked whether normality can be assumed, see Supplement 3. For the *t*-tests, effect size is reported using Cohen’s d, for the ANOVAs, partial η^2^. Finally, we analyzed whether participants’ views of sex workers as victims influenced their medical evaluation (hypothesis IV). To this end, we ran regression analyses with the estimated prevalence of various disorders (as described above) as dependent variable, the APPS subscale “sex workers as victimized vs. choosing” as predictor, and age and gender as covariates.

*p*-values below 0.05 were considered significant, with *p < 0.05, **p < 0.01, and ***p < 0.001.

### 2.5. Role of the funding source

The funder of the study had no role in study design, data collection, analysis, and interpretation, writing of the report, or in the decision to submit the paper for publication.

## 3. Results

### 3.1. Sample characterization

**Figure 1:**
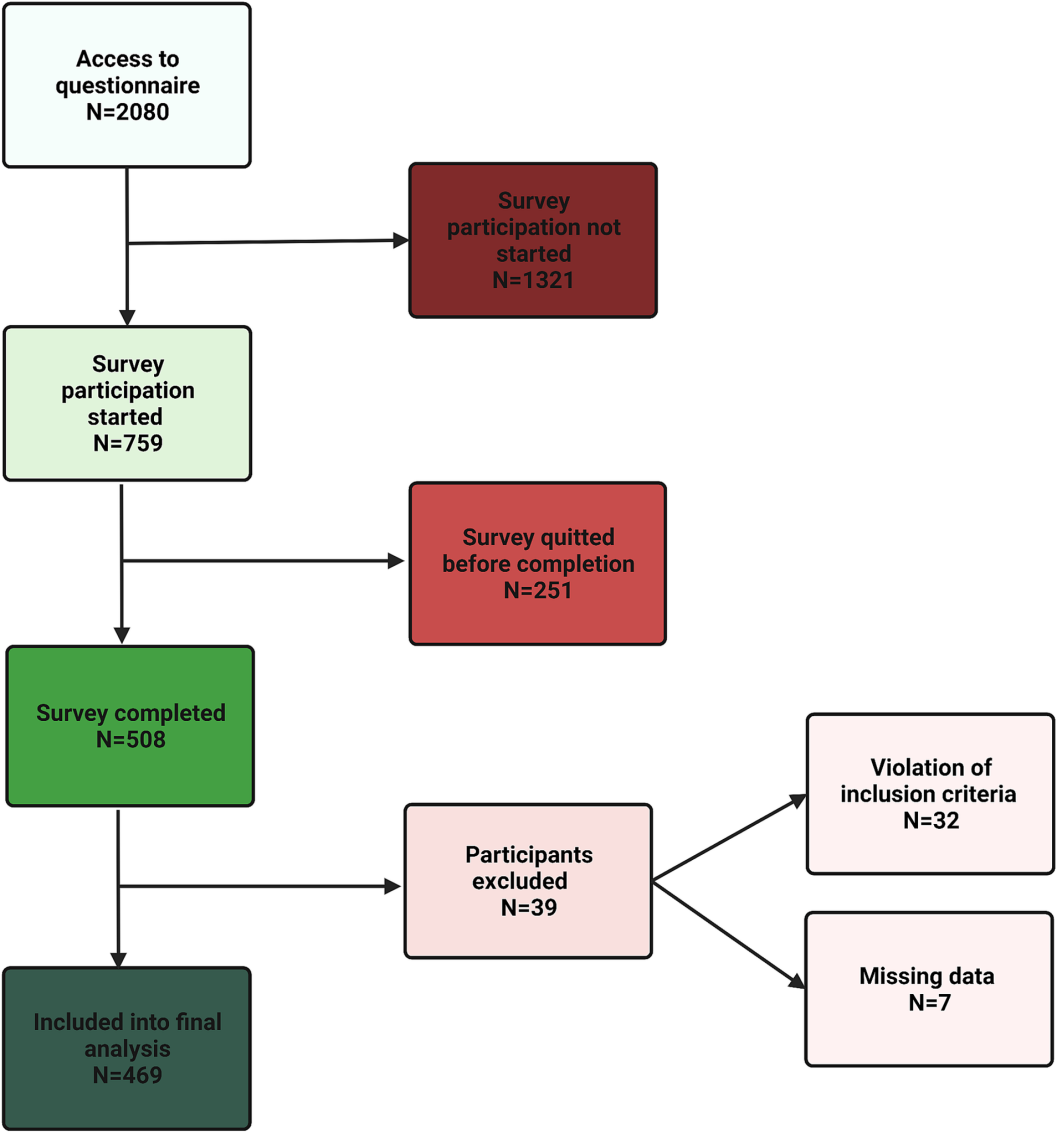
CONSORT (Consolidated Standards of Reporting Trials) diagram.

After data exclusion as described in the methods section, 469 participants were included into analysis as depicted in Figure 1. Participants’ characteristics are summarized in Table 1.

### 3.2. Treatment frequency of sex workers

Out of all participants, 20% indicated to treat one to ten sex workers per year, and a further 26.4% stated not to know the number of sex workers treated. Only 12% described frequent professional contacts with sex workers with ten or more per year. Three participants regarded being a professional sex worker as an exclusion criterion to take care of a sex worker patient and further four considered to refuse treatment “at least under specific circumstances.”

### 3.3. Attitudes towards prostitutes and prostitution scale

**Figure 2:**
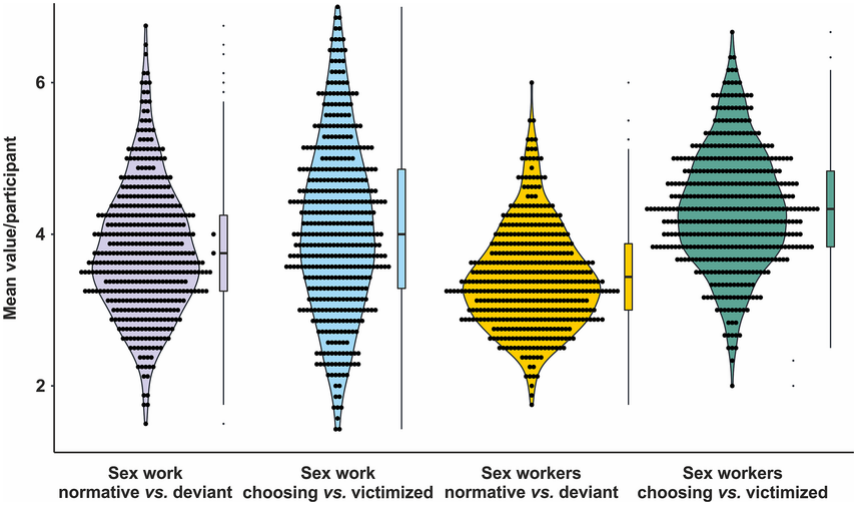
Attitudes towards sex workers measured with the four APPS subscales. Dots represent individual data points of the mean participants’ value of the respective APPS subscale according to a 7-point Likert scale (1 = do not agree at all, 7 = totally agree); summary statistics are provided as boxplots.

The 29-items of the APPS were analyzed according to the four underlying factors, respectively, subscales. On average, attitudes towards sex workers and sex work showed a remarkable variance, even though most participants reported values around the center of the subscales. Ranges and average attitudes are depicted in Figure 2.

### 3.4. Influence of age and gender

In a first step, we analyzed whether age and gender are associated with participants’ attitudes. We found a significant difference regarding the APPS subscale “sex work as choosing vs. victimized”, where women tended to rate sex workers as more victimized, *t*(225) = −2.02, *p* = 0.044 (uncorrected), even though the difference was small (mean (SD): 4.24 (0.76) for men and 4.40 (0.78) for women), *d* = −0.212. For the other three subscales, no significant gender associations were observed (all *p*-values *p* > 0.38).

For age, we found a significant correlation both with the “sex work as choosing vs. victimized” subscale, *r*(462) = 0.133, *p* = 0.004, and the “sex work as choice vs. victimization” subscale, *r*(462) = 0.182, *p* < 0.000, indicating that older participants tended to rate sex work as less of a choice and sex workers as more victimized. For the other two subscales, no significant correlation with age emerged (*p* = 0.23 and *p* = 0.75, respectively).

### 3.5. Influence of professional contact frequency with sex workers on attitudes towards them

**Figure 3:**
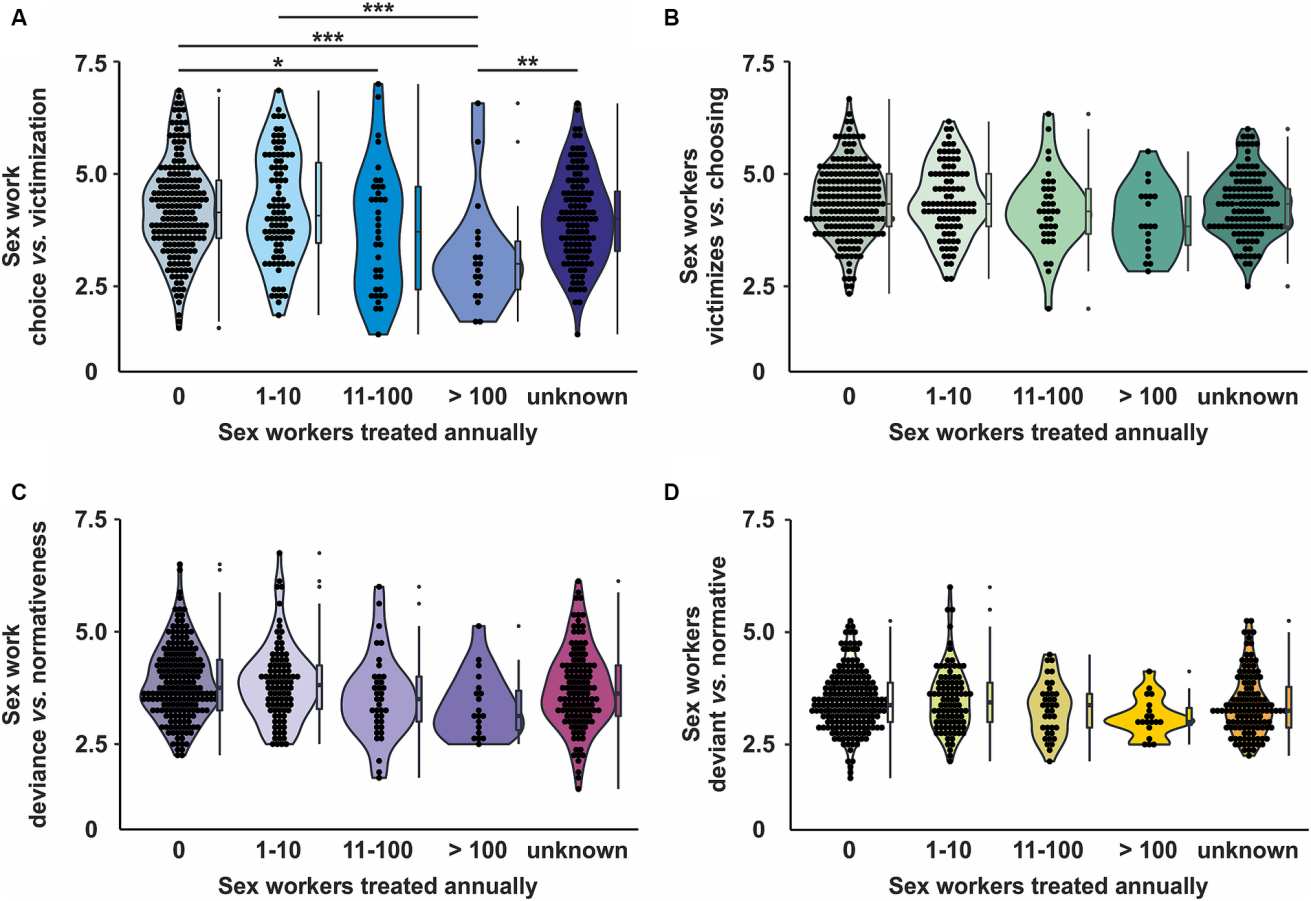
Influence of professional contact with sex workers on attitudes towards sex workers. **(A)** Influence on “Sex work as choice vs victimization” - subscale **(B)** Influence on “Sex workers as victims vs choice” - subscale **(C)** Sex work as deviance vs normativeness” - subscale **(D)** Influence on “Sex workers as deviant vs normative” - subscale. Dots represent individual data points; summary statistics are provided as boxplots. Significant results are indicated by asterisks (*p < 0.05, **p < 0.01, ***p < 0.001).

Following the contact hypothesis (showing that more contact with a stereotyped group reduces stereotypes), we expected attitudes to depend on the participants’ frequency of healthcare-related contacts with sex workers. We found a significant influence of contact frequency on the “sex work as victimized vs. choosing” subscale, *F* (4) = 5.296, *p* < 0.001, η^2^ = 0.07, and on the “sex work as deviance vs. normativeness” subscale of the APPS, *F* (4) = 2.782, *p* = 0.026. η^2^ = 0.03 (Figures 3A,C). The post-hoc test showed that those with more contact to sex workers regarded sex work to a lesser extent as a choice. However, no significant differences emerged in the post-hoc test regarding the “sex work as deviance vs. normativeness” subscale. Interestingly, contact with sex workers yielded neither a significant effect on the scales regarding sex workers themselves, nor on the “sex workers as victimized vs. choosing” subscale, *F* (4) = 1.790, *p* = 0.130, η^2^ = 0.03, or on the “sex workers as deviant vs. normative” subscale, *F* (4) = 2.146, *p* = 0.074, η^2^ = 0.02 (Figures 3B,D).

### 3.6. Influence of attitudes around victimization on medical evaluation

**Figure 4:**
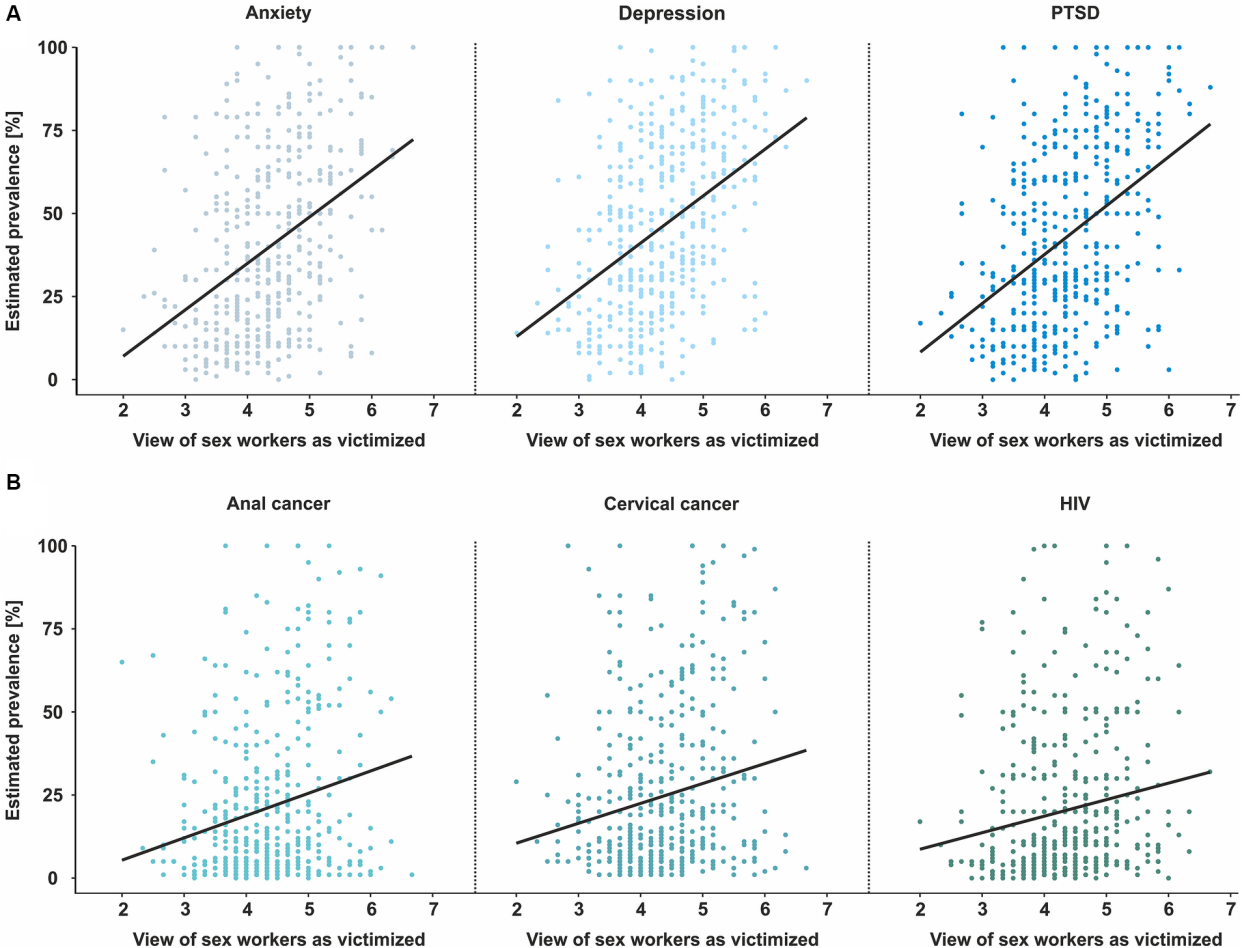
Healthcare providers’ estimations of disease prevalence in sex workers in relation to their attitudes. **(A)** Estimated prevalence of anxiety, depression, and post-traumatic stress disorder (PTSD). **(B)** Estimated prevalence of anal cancer, cervical cancer, and Human Immunodeficiency Virus (HIV) infection. Each dot represents the prevalence estimation of an individual participant; black lines depict the correlation between the rated attitude of the APPS subscale “sex workers as choice vs. victimization” in relation to the estimated prevalence.

**Figure 5:**
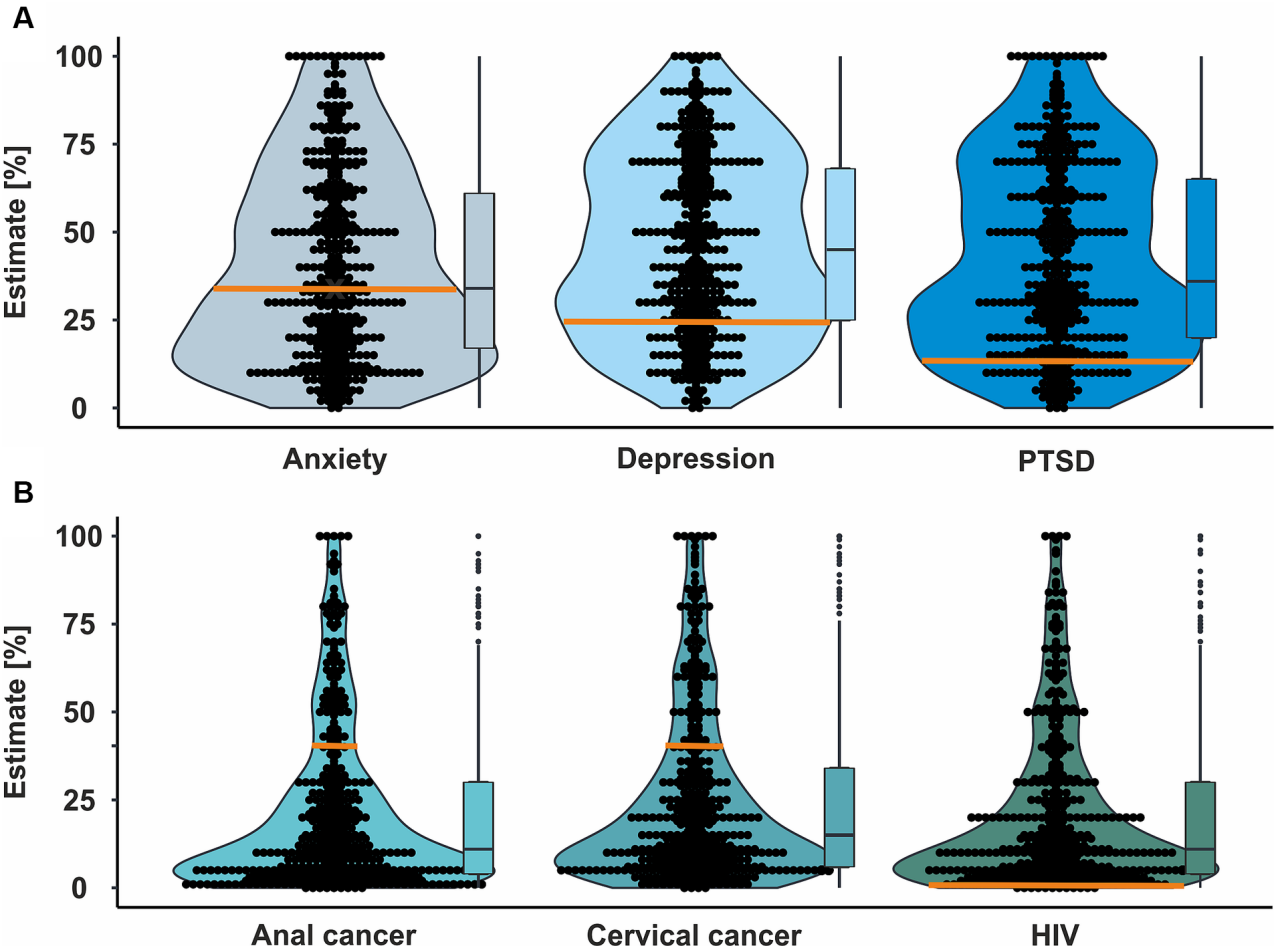
Estimation of prevalence in sex workers for different mental and somatic diseases by healthcare providers. **(A)** Estimation of prevalence for anxiety, depression, and post-traumatic stress disorder (PTSD). Orange lines represent the actual prevalence in the broader area of Zurich (Switzerland) as no detailed prevalence is reported for Germany (21) and both countries are reasonably similar. **(B)** Estimation of prevalence for anal cancer, cervical cancer, and Human Immunodeficiency Virus (HIV) infection. Each dot represents the prevalence estimation of an individual participant; summary statistics are provided as boxplots. The orange line represents the actual prevalence for HIV (22). As no data for prevalence of anal and cervical cancers in sex workers are available, the orange lines here represent Human Papilloma Virus (HPV) prevalence in sex workers in Europe (23) as an example. HPV is the most important causative agent for both types of cancer and thus, the prevalence of its risk factor cannot be lower than the actual prevalence of the diseases.

Next, we analyzed whether perceiving sex workers as victims is related to healthcare professionals’ medical evaluation. Indeed, all estimates of disease prevalence showed a significant connection between the “sex work as choosing vs. victimized” subscale of the APPS and the estimated point prevalence (Figure 4 and Table 2). This effect was substantially stronger for mental than for somatic disorders. In addition, the participants estimated high percentages of the prevalence for all disorders (see Figure 5).

**Table 2:** Regression coefficients for the influence of the “prostitutes as choosing vs. victimized” scale on the estimated prevalence of different medical conditions.

	*β*	*p*	*R* ^2^
PTSD	0.414	0.000	0.18
Anxiety	0.403	0.000	0.16
Depression	0.425	0.000	0.18
HIV	0.157	0.001	0.04
Anal cancer	0.175	0.000	0.04
Cervical cancer	0.203	0.000	0.05

*ß*, regression coefficient. *R*^2^ refers to the entire model, including age and gender.

Intuitively, healthcare professionals’ estimates of disease prevalence in sex workers should result from their expertise rather than their attitudes towards sex workers. Hence, we re-ran the analyses including participants’ profession and the interaction between their profession and the APPS-subscale as predictors. For neither of the prevalence estimates, we found a significant interaction between profession and the APPS-subscale (all *p*-values >0.05), indicating that the influence of participants’ attitudes on the prevalence estimated did not differ between distinct healthcare professions.

## 4. Discussion

Several studies have demonstrated a discrepancy between sex workers’ and other citizens’ utilization of healthcare services (4, 5, 24–26). However, little is known about the reasons. Prejudice of healthcare professionals against sex workers or even anticipated animosities by sex workers may explain the barriers for sex workers to utilize healthcare services at least in part. Yet, data about healthcare professionals’ knowledge of and attitudes towards sex workers has been largely missing. This study is the first to report such data for Germany.

Recently, a large population-based survey conducted in Germany assessed the attitudes of the general population towards sex workers (19). Although no formal statistical comparison between this survey and our study is possible based on the differences between questionnaires, similar aspects were evaluated. The overall results of the present study are mostly in line with the results from that survey: In our sample 45.8% of the respondents agreed (to some extend) to the statement “sex work is a job like any other” while in the general population 47% agreed to the statement “if a woman works as a prostitute by her own choosing, she has a job like any other to me.” Conversely, 55% of participants in our sample agreed (to some extend) to the statement “many sex workers are forced to do sex works” while 71% in the general population agreed to a similar statement. Finally, 89% of respondents stated that sex workers are at risk of suffering mentally because of their work compared to 69% in the sample representative for the general population. In conclusion, prejudice against sex workers seems to be as common in healthcare professionals as in the general population and was related to overestimation of prevalence for different diseases, especially mental diseases. Similar stigmatizing attitudes have been observed in healthcare professionals towards other stigmatized patient populations, e.g., patients with obesity (27, 28) or those affiliated to the *“*lesbian, gay, bisexual, transgender, queer +” (LGBTQ+) community (29, 30).

Analysing demographic factors in our sample, we found that women tended to regard sex workers as more victimized than men. This is in line with a study which was conducted in undergraduate psychology students (9). The study found that women were more likely than men to disagree with decriminalization and legalization and that women were more likely to view sex work as reflecting exploitation and subordination. At the same time, the study found no relation between the view of sex work as reflecting exploitation and subordination and a profeminist mindset (9). Hence, it is likely that other factors explain these gender differences. It may be that females are more empathic than male physicians (31). Another reason may be that women are more likely than men to have already experienced discrimination themselves (32). Both reasons may prompt women to sympathize more with sex workers than men.

Moreover, in the current study, older participants tended to rate sex work as less of a choice and sex workers as more victimized than younger participants. This conforms with a study from India which found less stigmatized attitudes towards sex work and sex workers in younger compared to older adults (33). A possible explanation might be that younger participants tend to be more liberal than older ones in their opinions (34).

Another study examined nursing students’ knowledge and attitude towards sex workers in Hong Kong. The study found that the attitude towards sex workers and the agreement with human rights were significantly associated with the willingness to care for sex workers. However, the students had little knowledge of sex workers and fittingly final-year students (who were assumed to have more knowledge) had more positive attitudes towards sex workers than first-year students (10). This might deliver a possible explanation for the results of the current study: Healthcare professionals’ attitudes towards sex work and workers might be close to that of the general population because they do not have substantially more knowledge about sex workers. Future studies will need to focus on the question whether healthcare training needs to implement education on the specific occupational health risks and needs of sex workers and possibly other marginal societal groups. This is of crucial importance since other studies have shown that stigma-related barriers hinder healthcare utilization of sex workers (35). Whether more information alone would suffice, however, is questionable. Indeed, factual information is often insufficient to change stereotypes (36). Additionally, stereotyping often serves an (albeit subconscious) purpose and can be seen as motivated reasoning: For example, it might protect a person’s general view of the world (e.g., the “just-world belief” (37)) or might serve as a way to stabilize one’s self-esteem (38). Thus, providing factual information can only be considered as a potential first step.

Remarkably, more than 60% of our participants stated to treat none or less than ten sex workers per year. In 2021, 23,700 sex workers were officially registered in Germany as compared to 40,400 pre-pandemic. The estimated number of unreported cases is up to ten times higher (19). Thus, we assume that healthcare professionals often simply did not know they were treating a sex worker. Whether the patients’ profession was not a subject during medical history taking or nescience was caused by non-disclosure of the sex worker due to anticipated stigmata remains elusive. However, knowledge of a sex worker’s profession is of particular interest since sex workers have a higher risk for numerous health conditions such as human papilloma virus (HPV)- infections (39), related cancers, and/or other sexually transmitted diseases (STI), and are likely to be underdiagnosed and -treated (40). Although no modified screening procedures are established for sex workers, individualized handling for screening and eventually vaccination strategies are desirable (41). Thus, the knowledge on patients’ profession and its specific risk factors adds another layer to personalized preventive measures. However, the flipside could be that disclosing one’s profession might lead to more overt discrimination: Three participants of our study stated that they would refuse to treat sex workers, and another four stated they would do so under certain circumstances. Although these extreme statements were very rare, they do illustrate how dramatic stigmatization of sex workers might be in the healthcare system.

Interestingly, participants estimated a surprisingly high prevalence for somatic and mental disorders in sex workers. Although the exact prevalence in relation to the professional context (and thus, sex work) is not captured within the existing epidemiological registries in Germany and remains therefore an estimate itself, we expect the estimates being highly overrated. For example, a median prevalence of 20.7% for HIV seems far too high given the high frequency of obligatory healthcare check-ups in sex workers. This is supported by the fact that a prevalence of 0.2% for HIV among sex workers in Germany in 2010 was indicated (22), although this prevalence may not have captured illegal sex workers. In addition, small data sets are published for mental disorders among sex workers in Switzerland: Rössler et al. (21) interviewed 193 sex workers in and around Zurich and found a one-year prevalence of 24% for major depression, 34% for anxiety disorder, and 13% for post-traumatic stress disorder. Thus, healthcare professionals should be careful not to overestimate sex worker’s health risks. Our data are in line with previous views from sex workers themselves who reported that some healthcare professionals immediately assume they must suffer from an STI, are traumatized, or regard their work as the sole cause of any mental health issues, ignoring both the patient’s free will and true motivation (42, 43). In addition, sex workers and their clients are legally bound by German law to practice adequate prevention of STIs by consistent use of condoms. Given these results, it seems likely that these wrong assumptions might guide against evidence-based treatment.

Contrary to our hypothesis, more frequent professional contact with sex workers was not clearly correlated to participants’ views of sex workers: Across all frequencies of contact, the evaluation of sex workers and sex work as morally deviant was relatively constant. Only the view of sex work as choice differed, and those with more contact rated sex work as more of a choice. Interestingly, however, this did not extend to the evaluation of sex workers, who were not seen as more choosing/less victimized. As of yet, the reasons for this are unclear. One possibility would be that participants’ evaluation of sex workers as victims vs. choosing agents is relatively stable, and therefore does not change by frequent contacts (unlike factually incorrect stereotypes that would change). Interestingly, healthcare professionals’ estimates of the prevalence of various disorders were not only strikingly incorrect at times, but they were also influenced by participants’ attitudes towards sex workers: If sex workers were seen as more of a victim, they were attributed a higher prevalence, particularly of mental disorders. This indicates that healthcare professionals might be influenced by prejudices rather than by objective facts they should have acquired during their professional education. Alternatively, there might be a knowledge gap concerning sex workers during training of healthcare professionals which might need improvement. In any case, this finding indicates that healthcare professionals might rely on their gut feeling rather than on profound knowledge when estimating medical risks for sex workers, with potentially detrimental effects.

While our study provides valuable first information, it should be noted that it represents data from a convenience sample. Additionally, while being a sufficiently large sample, it is relatively small compared to the study population: There are about 420,000 physicians in Germany (44), about 50,000 psychotherapists (45), about 485,000 nurses in hospitals and almost a million further nurses in ambulatory settings or care homes (46). Hence, the study sample is not representative for the total group of healthcare professionals. The demographic data of the respondents were skewed towards younger age and female gender. Moreover, the majority of respondents worked in a hospital and in cities of more than 100,000 inhabitants. Also, the group of medical students was overrepresented compared to other professional groups. Attitudes towards sex work and sex workers might therefore differ in a more representative group of healthcare personnel and possibly in specific subgroups. Similarly, the view of sex work and sex workers is influenced by cultural and regional specifics. For example, sex work is legal in Germany, and our data might therefore not generalize to other European countries (e.g., France). Future studies should try to examine these questions in a more representative sample. Moreover, the results from our study do not allow any casual inference. It can only be speculated that negative attitudes towards sex work and sex workers may contribute to a poorer medical treatment. Future prospective trials are needed to address this hypothesis.

In conclusion, our data provide first evidence that healthcare professionals’ attitudes towards sex work and sex workers are similar to that of the general population, and that these are at least partly influenced by sociodemographic factors. Our data indicate that many healthcare professionals are uncertain how many sex workers they treat annually. It remains, however, unanswered if the sex worker does not disclose his/her profession to avoid anticipated discrimination or if the profession was not a subject of the medical history. Crucially, participants’ attitudes influenced their medical evaluation, indicating that more education about specific health risks of sex workers might be necessary to provide stigmatization-free healthcare. If healthcare professionals create environments in which sex workers are willing to disclose their profession, and healthcare institutions disseminate more information about specific health risks, this might enable better and more personalized medical care.

## Data availability statement

The raw data supporting the conclusions of this article will be made available by the authors, without undue reservation.

## Ethics statement

The studies involving humans were approved by IRB, Medical Faculty of the University Duisburg-Essen, Germany, ethics vote no.: 22-10678-BO. The studies were conducted in accordance with the local legislation and institutional requirements. The participants provided their written informed consent to participate in this study.

## Author contributions

BL, AT, SR, and NN: conceptualization, formal analysis, funding acquisition, methodology, project administration, visualization, writing – original draft, and writing – review and editing. RV: data curation, methodology, and project administration. All authors contributed to the article and approved the submitted version.

## Funding

This study was supported by the Mercur Research Center Ruhr (Essen, Germany) as part of the “Global Young Faculty VII” program. The funder of the study had no role in study design, data collection, analysis, and interpretation, writing of the report, or in the decision to submit the paper for publication. NN and AT held a position that was funded in part by the German Research Foundation (DFG) and the University Medicine Essen Clinician Scientist Academy (UMEA; grant no. FU356/12-2). SR was funded by the DFG (grant no. RE4543/1-1).

## Conflict of interest

The authors declare that the research was conducted in the absence of any commercial or financial relationships that could be construed as a potential conflict of interest.

## Publisher’s note

All claims expressed in this article are solely those of the authors and do not necessarily represent those of their affiliated organizations, or those of the publisher, the editors and the reviewers. Any product that may be evaluated in this article, or claim that may be made by its manufacturer, is not guaranteed or endorsed by the publisher.
