# Supplementary material for: Attitudes towards sex workers: a nationwide cross-sectional survey among German healthcare providers
Source: Front Public Health. 2023 Sep 6;11:1228316. doi: 10.3389/fpubh.2023.1228316 (PMC10513093; doi:10.3389/fpubh.2023.1228316)
Supplement: Supplementary file 1 [file Data_Sheet_1.PDF]

Supplementary Material

Attitudes towards sex workers: A nationwide cross-sectional survey among German healthcare providers

Benedikt P. Langenbach[1,2*], Andreas Thieme[1-3*], Raquel van der Veen [2,3], Sabrina Reinehr[4#], Nina R. Neuendorff[5#]

[1]Department of Psychiatry and Psychotherapy, LVR University Hospital Essen, Faculty of Medicine, University of Duisburg-Essen, Virchowstr. 174, 45147 Essen, Germany

[2]Laboratory Center for Translational Neuro- and Behavioral Sciences (C-TNBS), University Hospital Essen, University of Duisburg-Essen, Hufelandstr. 55, 45147 Essen, Germany

[3]Department of Neurology, University Hospital Essen, University of Duisburg-Essen, Hufelandstr. 55, 45147 Essen, Germany

[4]Experimental Eye Research Institute, University Eye Hospital, Ruhr-University Bochum, In der Schornau 23-25, 44892 Bochum, Germany

[5]Department of Haematology and Stem Cell Transplantation, University Hospital Essen, Faculty of Medicine, University of Duisburg-Essen, Hufelandstr. 55, 45147 Essen, Germany

* These authors contributed equally to this work and share first authorship
# These authors contributed equally to this work and share last authorship

*** Correspondence:**
Nina Rosa Neuendorff, MD
nina.neuendorff@uk-essen.de

1 German translation of the Attitudes towards Prostitutes and Prostitution Scale (APPS)

Bitte beantworten Sie folgende Aussagen von

1=stimme überhaupt nicht zu bis 7=stimme sehr zu.

1) Prostitution ist Menschenhandel.
2) Die meisten Prostituierten sind drogenabhängig.
3) Prostitution erzwingt ungewolltes Sexualverhalten.
4) Prostitution ist wichtig, um jugendliche Männer über Sexualität aufzuklären.
5) Prostituierte verdienen viel Geld.
6) Prostitution ermöglicht es den Frauen, die sie ausüben, ihre sexuellen Fantasien auszuleben.
7) Prostitution erhöht den gesellschaftlichen Konsum von Drogen.
8) Die meisten Prostituierten sind moralisch verdorben.
9) Ohne Prostitution würden mehr Frauen vergewaltigt.

10) Die meisten Prostituierten sind hässlich.
11) Prostitution schadet der Gesellschaftsmoral.
12) Prostituierte verbreiten AIDS.
13) Prostitution ist eine Verletzung der Menschenwürde von Frauen.
14) Prostituierte genießen die Kontrolle von Männern.
15) Frauen werden Prostituierte, weil sie keine richtige Ausbildung haben.
16) Prostitution gibt Männern eine Möglichkeit, Stress abzubauen.
17) Prostitution ist eine Form von Gewalt an Frauen.
18) Prostituierte mögen Sex.
19) Viele Prostituierte sind Studentinnen, die einen bequemen, einträglichen Job haben wollen.
20) Prostituierte sind Opfer von Drogenmissbrauch.
21) Für einige Frauen ist Prostitution ein Weg, Macht und Kontrolle zu erlangen.
22) Frauen entscheiden sich dafür, Prostituierte zu werden.
23) Prostitution erhöht die Raten an sexuell übertragbaren Krankheiten.
24) Prostitution ist eine Form von Vergewaltigung, bei der das Opfer bezahlt wird.
25) Prostitution schadet der Institution Ehe.
26) Die meisten Prostituierten arbeiten nur einige Jahre als Prostituierte, um sich finanziell abzusichern.
27) Prostituierte sind unfähig, ihre Situation zu verlassen.
28) Prostitution ist ein Weg, ökonomisch benachteiligte Bevölkerungsgruppen zu stärken.
29) Durch Prostitution können schöne Frauen einen Ehemann finden.

2 Additional items

1) Sex work is a job like any other
2) Many sex workers are forced to do sex works
3) Sex workers are at risk of suffering mentally because of their work

3 Density plots and Q-Q-plots for the four subscales of the ATPPS

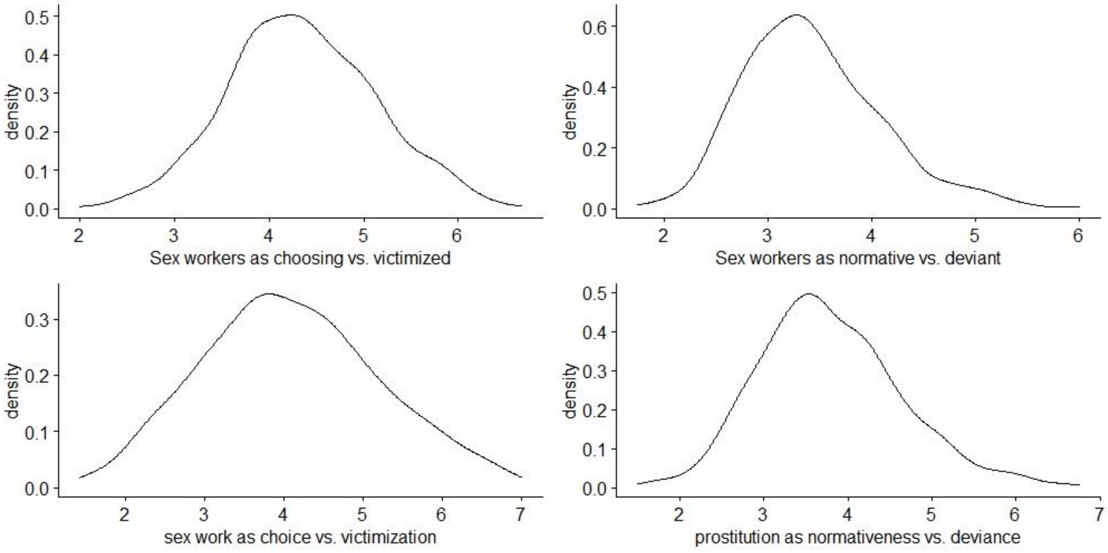

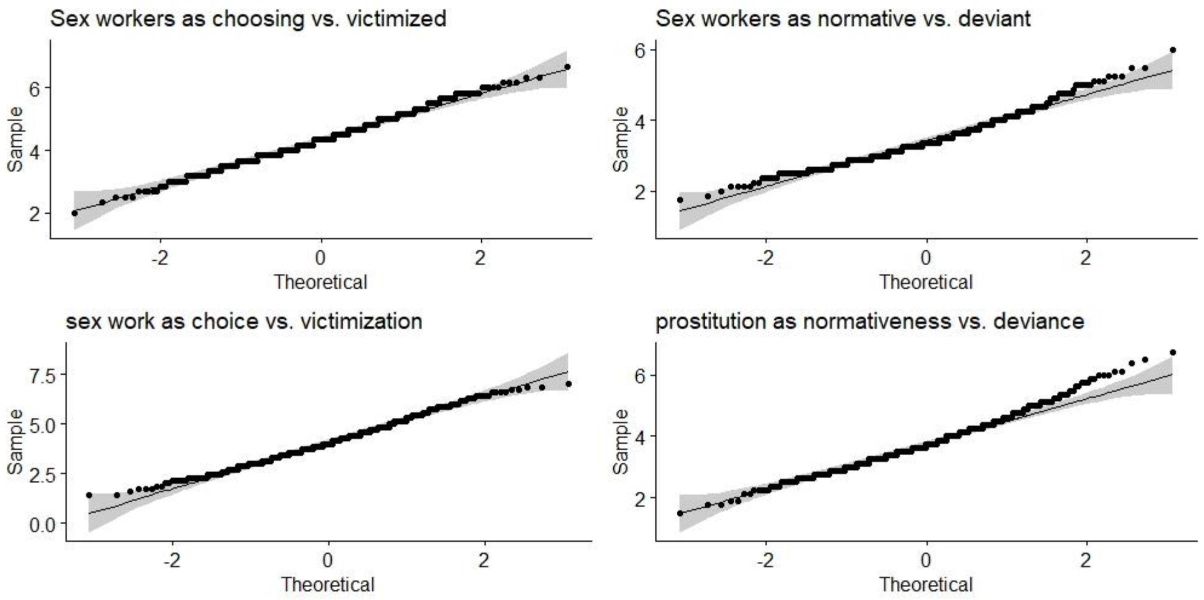
Sex workers as choosing vs. victimized
Sample
Theoretical
Sex workers as normative vs. deviant
Sample
Theoretical
sex work as choice vs. victimization
Sample
Theoretical
prostitution as normativeness vs. deviance
Sample
Theoretical
